# Tri-Layered Doxycycline-, Collagen- and Bupivacaine-Loaded Poly(lactic-*co*-glycolic acid) Nanofibrous Scaffolds for Tendon Rupture Repair

**DOI:** 10.3390/polym14132659

**Published:** 2022-06-29

**Authors:** Yi-Hsun Yu, Shih-Jyun Shen, Yung-Heng Hsu, Ying-Chao Chou, Ping-Chun Yu, Shih-Jung Liu

**Affiliations:** 1 Bone and Joint Research Center, Department of Orthopedic Surgery, Chang Gung Memorial Hospital at Linkou, Taoyuan 33305, Taiwan; alanyu1007@gmail.com (Y.-H.Y.); laurencehsu.hsu@gmail.com (Y.-H.H.); enjoycu@ms22.hinet.net (Y.-C.C.); 2 Department of Anesthesiology, Chang Gung Memorial Hospital at Linkou, Taoyuan 33305, Taiwan; m8420@cgmh.org.tw; 3 Department of Mechanical Engineering, Chang Gung University, Taoyuan 33302, Taiwan; kfv012003@gmail.com

**Keywords:** Achilles tendon rupture, poly(lactic-*co*-glycolic acid), doxycycline, bupivacaine, electrospinning, nanofibers, tissue engineering scaffold

## Abstract

Achilles tendon rupture is a severe injury, and its optimal therapy remains controversial. Tissue engineering scaffolds play a significant role in tendon healing and tissue regeneration. In this study, we developed tri-layered doxycycline/collagen/bupivacaine (DCB)-composite nanofibrous scaffolds to repair injured Achilles tendons. Doxycycline, collagen, and bupivacaine were integrated into poly(lactic-*co*-glycolic acid) (PLGA) nanofibrous membranes, layer by layer, using an electrospinning technique as healing promoters, a 3D scaffold, and painkillers, respectively. After spinning, the properties of the nanofibrous scaffolds were characterized. In vitro drug discharge behavior was also evaluated. Furthermore, the effectiveness of the DCB–PLGA-composite nanofibers in repairing ruptured Achilles tendons was investigated in an animal tendon model with histological analyses. The experimental results show that, compared to the pristine PLGA nanofibers, the biomolecule-loaded nanofibers exhibited smaller fiber size distribution and an enhanced hydrophilicity. The DCB-composite nanofibers provided a sustained release of doxycycline and bupivacaine for over 28 days in vivo. Additionally, Achilles tendons repaired using DCB-composite nanofibers exhibited a significantly higher maximum load-to-failure than normal tendons, suggesting that the biomolecule-incorporated nanofibers are promising scaffolds for repairing Achilles tendons.

## 1. Introduction

Tendons are soft tissues that link muscles to bones, enabling passive and active joint movement during force transmission. They have a complex hierarchical structure of collagen (mainly type I) and elastin embedded in a proteoglycan matrix [1,2]. Collagen type I is stiff, elastic and provides mechanical strength and durability. [3] Most large tendons, including the Achilles tendon, rotator cuff, forearm extensor, and flexor tendons, are susceptible to overuse, resulting in tendinopathy [4,5]. Acute tendon injury, involving partial or total rupture, damages tendon integrity and leads to a loss of strength and movement. The treatment of acute tendon injury includes conservative management and surgical repair. During the healing process, a stochastically oriented preliminary network of collagen type III is established at the injury site as scar tissue [6]. Nevertheless, tendons are very slow to heal, requiring a much longer time to produce collagen type I fibrils than the rest of the musculoskeletal system. Consequently, the wounded tendon loses its elasticity and elongation until collagen type III is replaced by collagen type I. Aging or repetitive tendon injury can enhance the ratio of collagen type III/type I in tendons [7,8,9].

Despite progress in modern medicine, the treatment of ruptured tendons remains a challenge. Various methods, such as ultrasound therapy, platelet-rich plasma injection, local or systemic drug administration, and the supplementation of growth factors targeted at the injured site, have been proposed to treat Achilles tendon rupture [10,11,12,13,14,15,16,17]. Healing efficacy is a key factor to determine the success of tendon repair. With advances in biomaterial science, tissue engineering has provided promising strategies for treating and replacing injured organs [18]. Scaffolds play a significant role in tendon healing and tissue regeneration. They can link and assist cells and tissues, regulating distinct functional activities of cells [19]. In addition, the biological functionality of the scaffolds, including biocompatibility and cell differentiation, can be optimized through nanotechnology. Three-dimensional nanoscale vehicles can transport active biomolecules to injured tissues and promote the preservation, proliferation, and differentiation of normal cells. An ideal scaffold for tendon repair should mimic the native extracellular matrix (ECM) and support cell adhesion, proliferation, and maturation. Scaffold material should be non-toxic and biocompatible. It should be resorbable and able to gradually biodegrade and metabolize in the body as cells proliferate. Excellent processing properties are also essential for the scaffold as it should be easily fabricated into the desired geometry with a porous structure and suitable pore size. Electrospinning is a versatile and efficient method for the fabrication of nanostructured fibers. Nanofibrous scaffolds made of biodegradable and biocompatible polymers have received increasing attention due to their superior flexibility, efficacy, and extraordinary physiochemical characteristics, such as a large surface area, small diameter, gas permeability, and excessive aspect ratio [20]. In addition, the similarity of the nanofibrous structure of the scaffolds with that of the ECM facilitates cell attachment and proliferation, making it a great candidate for various medical applications [21].

Doxycycline is an antibiotic medicine that shows experimental and clinical efficacy in treating tendon rupture and a feasibility for local applications during surgery or systemic administration [14,22,23]. Collagen is the main structural protein of most tissues in the human body. It also plays an important role in maintaining the biological and structural integrity of the ECM and regulating the morphology, adhesion, migration and differentiation of cells, making this natural polymer a promising biomaterial for scaffolds in tissue regeneration [24]. Bupivacaine is an effective painkiller with special features from its amide group and has been used as a local, epidural, and spinal anesthesia, and to regionally infiltrate injured areas [25].

Biodegradable polymers, such as polyglycolide (PGA), polylactide (PLA), and polycaprolactone (PCL), are used in vitro and in vivo in various medical fields [18,19,21,25]. Poly(lactic-*co*-glycolic acid) (PLGA), the copolymer of PGA and PLA, has been intensively studied because it can induce favorable tissue integration, exhibit a degradation rate similar to that of native collagen matrices, and deliver loaded drugs to the target site [26,27,28].

In this study, we developed doxycycline/collagen/bupivacaine (DCB) PLGA nanofibrous scaffolds to repair injured Achilles tendons. An electrospinning technique was employed to integrate doxycycline, collage, and bupivacaine layer by layer into PLGA nanofibrous membranes as healing promoters, 3D scaffolds, and painkillers, respectively. The properties of the prepared nanofibrous mats were characterized after spinning. The in vivo and in vitro drug release behavior was evaluated. In addition, the efficacy of the electrospun DCB-composite nanofibers in repairing ruptured Achilles tendons was investigated using a rat tendon model. Finally, a histological analysis was performed.

## 2. Materials and Methods

### 2.1. Materials and Fabrication of Biomolecule-Loaded PLGA Nanofibers

The polymeric material was a commercially available PLGA polymer (lactide/glycolide: 50/50 with a molecular weight of 24,000–33,000 Da, Resomer RG 503; Sigma-Aldrich, Saint Louis, MO, USA). The biomolecules, including doxycycline, collagen type I, and bupivacaine, were purchased from Sigma-Aldrich (St. Louis, MO, USA). The solvent, 1,1,1,3,3,3-hexafluoro-2-propanol (HFIP), was acquired from Sigma-Aldrich (St. Louis, MO, USA).

Tri-layered doxycycline-, collagen- and bupivacaine-loaded nanofibrous scaffolds were prepared by electrospinning technique. To fabricate the doxycycline-loaded nanofibrous layer, doxycycline (280 mg) and PLGA (1,120 mg) were mixed with HFIP (5 mL). The mixture was subsequently electrospun into nanofibers by a lab-scale setup involving a syringe/needle (internal diameter: 0.42 mm), a high-voltage supply (DC voltages: 36 kV; currents: 4.16 mA/125 W), and a grounded collector. For the preparation of collagen/PLGA and bupivacaine/PLGA nanofibrous layers, collagen (0.2 mL), PLGA (1,120 mg), bupivacaine (280 mg), and PLGA (1,120 mg) were separately dissolved in HFIP (5 mL) and electrospun into nanofibers. The distance between the collector plate and needle tip was 15 cm, the voltage used was 17 kV, and the delivery speed of the solutions was 0.7 mL/h. All procedures were performed at room temperature (27 °C) and 70% humidity. Tri-layered DCB nanofibrous mats with a thickness of approximately 200 µm were then acquired. The fabricated biomolecule–PLGA nanofibers were subsequently incubated in a vacuum oven at 40 °C for 3 days to vaporize the HFIP.

### 2.2. Characterization of Integrated PLGA Nanofibers

#### 2.2.1. Scanning Electron Microscopy

The morphological structure of the electrospun biomolecule-loaded PLGA nanofibrous scaffolds was analyzed using a JSM–7500F scanning electron microscope (SEM; Joel, Tokyo, Japan) after coating with gold. The size distribution of the target nanofibers was defined using an image analysis program (ImageJ software, National Institutes of Health, Bethesda, MD, USA).

In addition, the density of the nanofiber was determined by dividing the mass by volume. The porosity of the nanofibrous scaffold was calculated as follows:

(1) $$Pore(\%)=\left\{1-\frac{\rho_{membrane}}{\rho_{polymer}}\right\}$$

where *ρ_membrane_* and *ρ_polymer_* represent the densities of the biomolecule–PLGA and pristine PLGA nanofibers, respectively.

#### 2.2.2. Wetting Angles

To determine hydrophilicity, the wetting angles of the nanofibers were assessed using a water contact angle analyzer (First Ten Angstroms, Portsmouth, VA, USA). Electrospun PLGA nanofibers (10 × 10 mm) were placed on the testing plate, and distilled water was carefully dropped on their surfaces. The wetting angles were then analyzed using a camera.

#### 2.2.3. Differential Scanning Calorimetry Assessment

The thermal behavior of pristine PLGA and biomolecule–PLGA nanofibrous scaffolds was measured using differential scanning calorimetry (DSC) (TA Instruments, New Castle, DE, USA). The samples were scanned from 30 °C to 300 °C and the heating rate was maintained at 10 °C/min.

#### 2.2.4. Tensile Strengths of Electrospun Nanofibrous Scaffolds

The tensile strength of the electrospun nanofibrous scaffold was evaluated using a Lloyd tester (Ametek, Berwyn, PA, USA) equipped with a 2.5 kN load cell. Specimens (2 × 5 cm) were cut from the spun membranes for the experiments. The extension rate was maintained at 60 mm/min, and the stress–strain curves were monitored.

### 2.3. In Vitro Drug Elution of PLGA Nanofibers

An elution scheme was utilized to quantify the in vitro release profiles of doxycycline and bupivacaine from the PLGA nanofibers. Nanofiber samples were immersed in a medium composed of phosphate buffer (0.15 mol/L, pH 7.4) prior to quantification. First, a nanofibrous mat with a controlled size (1 cm × 1 cm, approximately 15–20 mg) was sliced and incubated in a test tube containing 1 mL of phosphate-buffered saline at a constant temperature (37 °C) for 24 h. The phosphate buffer (1 mL) was replaced every 24 h until the mat was completely solvated. The concentration of drugs in the buffer was assessed using a Hitachi L-2200 high-performance liquid chromatography (HPLC) system (Tokyo, Japan).

### 2.4. Experimental Model of Achilles Tendon Injury and Repair

Sprague Dawley (SD) rats, each weighing approximately 250 ± 25 g, were used for in vivo experiments. All animal studies were approved by the Institutional Animal Care and Use Committee of Chang Gung University (IACUC Approval No.: CGU108-120), and all animals were handled in accordance with the guidelines and regulations of the Ministry of Health and Welfare of Taiwan. The animals were sedated under general anesthesia in a polymethylmethacrylate chamber (40 cm× 20 cm× 28 cm) using isoflurane. Inhalational anesthesia was maintained throughout the operational process. For analgesia and hemostasis, the rats were injected with 0.5 mL 2% xylestesin-A and epinephrine mixture (1:100,000) in the right leg before the surgical procedure. The right leg was shaved and prepared using a standard antiseptic procedure. A 3 cm longitudinal cut lateral to the Achilles tendon was created on the skin. The Achilles tendon was identified by anatomizing the subcutaneous fat and surrounding soft tissues. The central portion of the Achilles tendon was transected and sealed end-to-end using a 5-0 Vicryl suture (Johnson & Johnson, New Brunswick, NJ, USA).

The rats were arbitrarily divided into three groups (normal, control, and DCB-composite nanofiber group). The animals in the normal group did not have any surgery, whereas the rats in the control group underwent surgery without any implantation. For the rats in the DBC nanofiber group, the Achilles tendons were circumferentially wrapped with DCB nanofibrous mats (1 cm × 1 cm) after the surgery. The injury was sealed with a 3-0 nylon suture (Johnson & Johnson, New Brunswick, NJ, USA). Bactericidal ointment was topically applied above the surgical wound to prevent infection. The rats were returned to their cages after complete recovery from anesthesia.

### 2.5. Bioactivities Examination

After the operation, the level of activity of the rat was monitored daily by keeping each rat in a lab-assembled animal behavior cage (ABC, 50 cm × 50 cm × 50 cm) for seven days. Nine sensors (HP100-A1, Azbil Corp., Tokyo, Japan) located at the top of the cage were used to monitor rat movements. Each sensor was triggered to record movement when the rat migrated from one area to another. The total triggered counts were recorded using a microprocessor and acquisition interface for seven consecutive days. Daily water and food intake were also recorded. Constant temperature (22 °C–25 °C), pressure (1 atm), and humidity (60–70%) levels were maintained for the entire observation period. After activity examination, the rats were returned to their initial cages for animal care.

### 2.6. In Vivo Drug Elution Characterization

To evaluate the in vivo elution behavior of DCB-composite nanofibers, tissues surrounding the DCB-wrapped Achilles tendon were sampled on days 1, 3, 7, 14, 21, and 28 post-implantation with a surgical procedure similar to that for implantation. The drug concentrations in the collected samples were subsequently evaluated using HPLC.

### 2.7. Specimen Assessments

#### 2.7.1. Gross Specimen Assessment

Tendons for evaluation were excised after the rats were euthanized. The cross-sectional diameters of the tendons where transection was performed were measured and recorded using a digital caliper.

#### 2.7.2. Mechanical Property Assessment

The retrieved Achilles tendons at 8 weeks post-implantation were evaluated using a Lloyd tensiometer with a 2.5 kN load cell (Ametek, Berwyn, PA, USA). The tendons were first wrapped with gauze and submerged in a saline solution to mimic the in vivo environment. The Achilles tendon was stretched at a rate of 60 mm/min during testing.

#### 2.7.3. Histology and Immunohistochemistry (IHC) Assay

Wet tissues obtained from the investigated animals were trimmed and dehydrated using a serial alcohol solution. The dehydration sequence was 70% ethanol (15 min), 90% ethanol (15 min), 100% ethanol (15 min), 100% ethanol (15 min), 100% ethanol (20 min), 100% ethanol (30 min), and xylene (overnight) using a Shandon Excelsior (Thermo Scientific, Altrincham, UK). Tissue sections (3–5 μm) were collected using a microtome (Sakura Finetek, Tokyo, Japan) for histological assessment. Furthermore, the acquired specimens were stained with hematoxylin and eosin (H&E), and the tenocytes and tissues were examined under a microscope with a magnification of 400.

Immunohistochemistry (IHC) staining was performed on 4 μm paraffin sections of tendon tissues to document the expression of the bone morphogenetic protein (BMP-2), vascular endothelial growth factor (VEGF), von Willerdrand factor (vWF), transforming growth factor beta (TGF-b), and type I and III collagens using a BOND-MAX Fully Automated IHC and ISH Staining System (Leica, USA). The antibodies used for IHC analyses included BMP-2 (polyclonal, 1:50, A0231, ABclonal, MA, USA), VEGF (polyclonal, 1:100, A0280, ABclonal, MA, USA), vWF (Picoband™ antibody, 1:200, PB9062, Boster Biological Technology, Pleasanton, CA, USA), TGF-β (polyclonal, 1:50, A2561, ABclonal, MA, USA), type I collagen (1:2000, A1352, ABclonal, MA, US), and collagen III (1:400, A00788-3, ABclonal, MA, US). Heat-induced epitope retrieval was accomplished (95 °C/30 min) in a citrate buffer (pH = 6.0) after deparaffinization and rehydration. The slides were then incubated with a hydrogen peroxide solution (3%) for 5 min. After washing with the supplied buffer, tissue sections were repaired for 40 min using ethylenediaminetetraacetic acid. This was followed by incubating the slides with the primary antibody for 60 min at 37 °C and overnight at 4 °C. After three rinses in buffer, the slides were incubated with secondary antibodies. Tissue staining was performed using a 3,3′-diaminobenzidine substrate chromogen solution. To measure the collagen I and III in the specimens, the average optical density was processed using ImageJ software (National Institutes of Health, Bethesda, MD, USA).

### 2.8. Statistical Analyses

Descriptive statistics are shown as the mean ± standard deviation. Data were analyzed using paired *t*-tests, and differences were considered statistically significant at *p* < 0.05.

## 3. Results

### 3.1. Characterization of DCB PLGA Nanofibers

**Figure 1:**
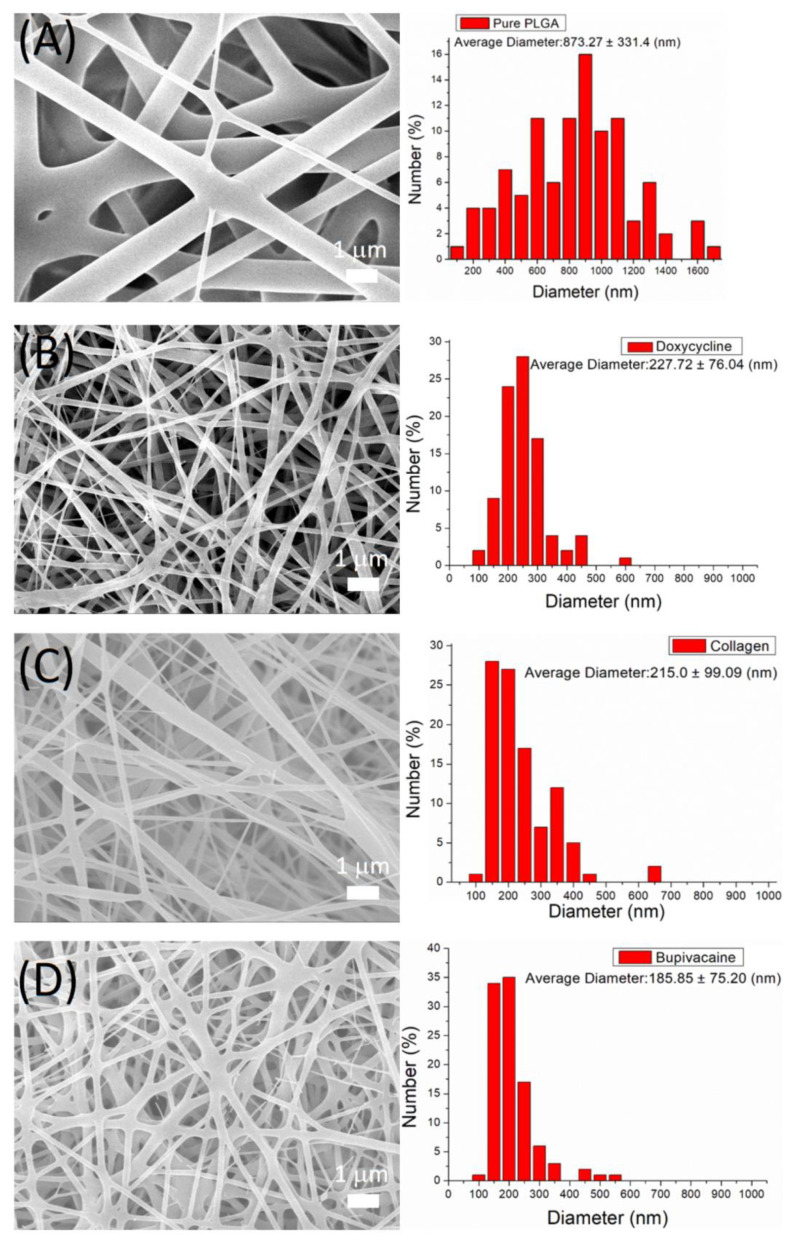
SEM image and size distribution of (**A**) pristine PLGA nanofibers, (**B**) doxycycline-loaded nanofibers, (**C**) collagen-loaded nanofibers, and (**D**) bupivacaine-loaded nanofibers.

The fabricated nanofibers were characterized by field-emission SEM under 10,000× magnification (Figure 1). The average diameters of electrospun nanofibers were 873.3 ± 331.4 (pristine PLGA), 227.7 ± 76.0 (doxycycline–PLGA), 215.0 ± 99.1 (collagen-PLGA), and 185.9 ± 75.2 nm (bupivacaine–PLGA). With the incorporation of biomolecules and the decreased percentage of PLGA, the diameter of the spun nanofibers was reduced because the nanofiber could be easily extended by the external electrical force. Compared with the pristine PLGA, the biomolecule-loaded nanofibers exhibited inferior fiber size distributions. The average porosity of the pristine PLGA nanofibers was 75.7%. With the loading of doxycycline, collagen, and bupivacaine, the porosities of the nanofibers increased to 83.6, 99.1%, and 83.3%, respectively.

**Figure 2:**
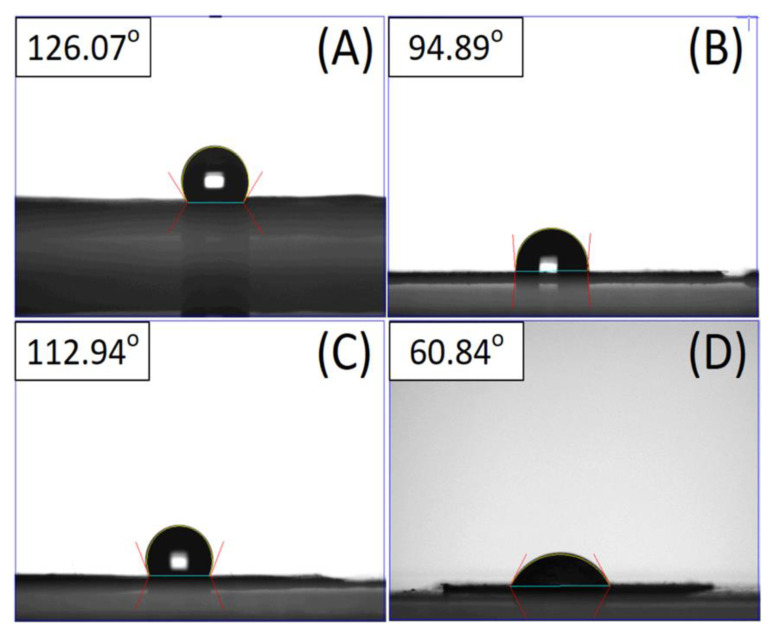
Wetting angles of (**A**) pristine PLGA nanofibers, (**B**) doxycycline-loaded nanofibers, (**C**) collagen-loaded nanofibers, and (**D**) bupivacaine-loaded nanofibers.

The water contact angles for the pristine and biomolecule-loaded PLGA nanofibrous mats were measured. Figure 2 shows that the contact angle of the pristine PLGA is 123.36°. The hydrophilicity was enhanced with the incorporation of doxycycline, collagen, and bupivacaine, and their contact angles were measured as 94.89°, 112.94°, and 60.84°, respectively.

**Figure 3:**
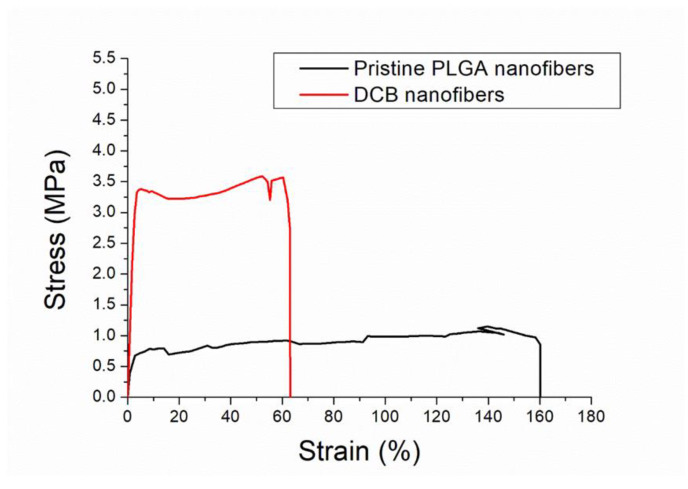
Stress–strain curves of pristine PLGA and DCB–PLGA-composite nanofibers.

The stress–strain curves of the spun nanofibrous mats were measured. The empirical data in Figure 3 illustrate that the DCB-composite nanofibers have a higher tensile strength and lower elongation at break than the pristine PLGA nanofibers.

**Figure 4:**
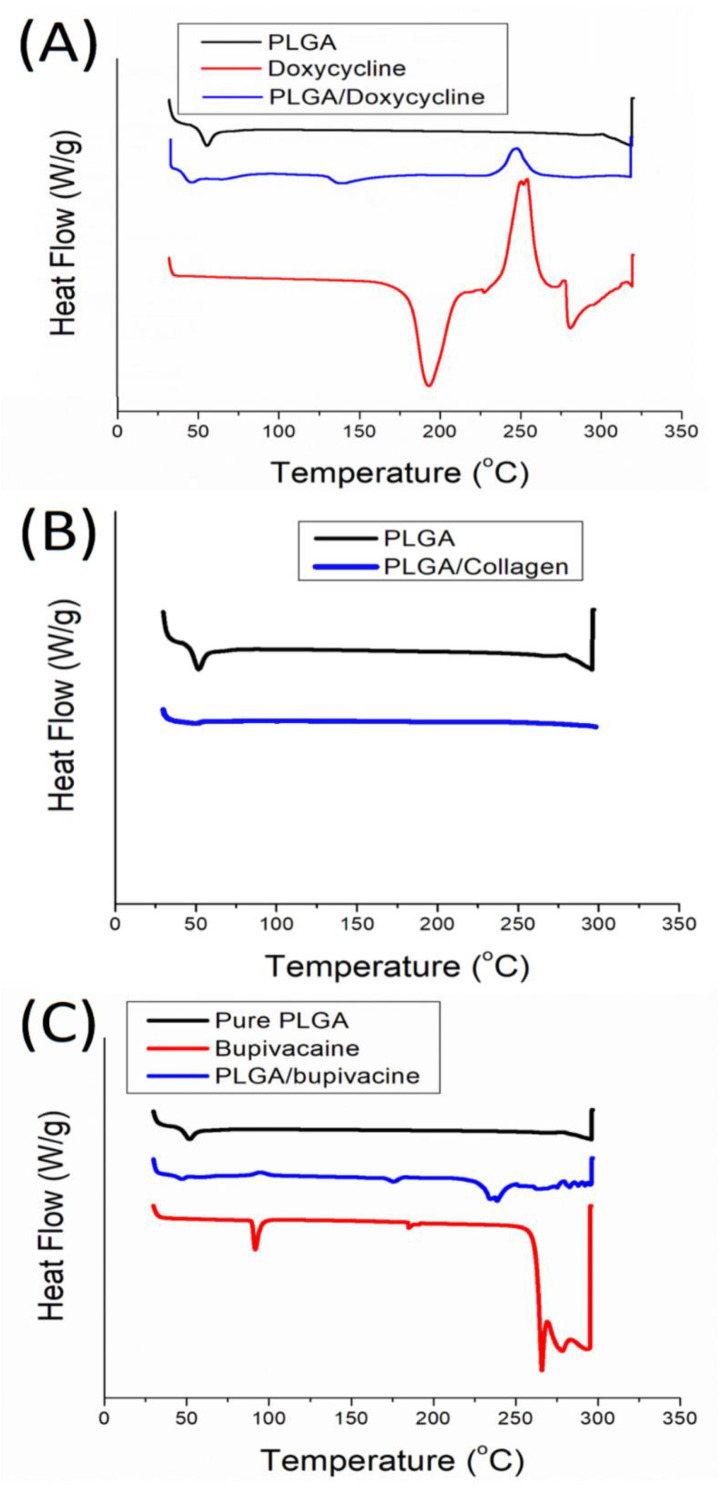
Thermograms of (**A**) doxycycline-loaded nanofibers, (**B**) collagen-loaded nanofibers, and (**C**) bupivacaine-loaded nanofibers.

Figure 4 compares the thermal behaviors of the pristine PLGA and biomolecule-loaded PLGA nanofibrous mats. After doxycycline was loaded in the PLGA matrix, the endothermal peak at 181 °C vanished [29], and the exothermal peak near 237 °C declined (Figure 4A). In Figure 4B, the glass transitional peak of PLGA near 52 °C almost disappeared after the incorporation of collagen, while in Figure 4C, the peaks of bupivacaine at 91.6 °C and 265.8 °C almost diminished after being included in the bulk PLGA [30]. These results confirm the successful incorporation of doxycycline, collagen and bupivacaine into the PLGA nanofibrous scaffolds.

### 3.2. In Vitro Drug Elution of Pharmaceuticals-Loaded PLGA Nanofibers

**Figure 5:**
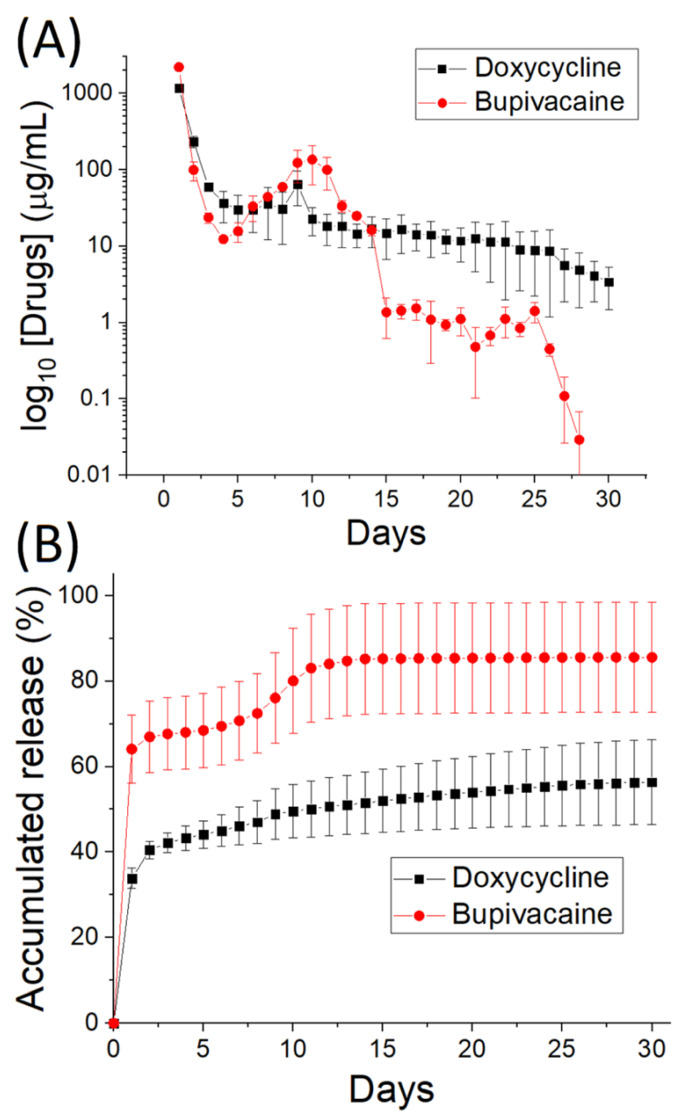
In vitro (**A**) daily and (**B**) cumulative release of doxycycline- and bupivacaine-loaded PLGA nanofibers.

The in vitro daily and cumulative drug-eluting quantifications of the electrospun doxycycline- and bupivacaine-loaded PLGA nanofibers are illustrated in Figure 5A,B, respectively. A preliminary elution peak for both drugs was observed on day one, whereas a second peak was observed on day 7. Thereafter, the drug release gradually decreased. Overall, the nanofibers provided a sustainable release of doxycycline and bupivacaine for 30 days, with approximately 55% and 82% of the loaded doxycycline and bupivacaine being discharged, respectively.

### 3.3. In Vivo Drug Elution Characterization

**Figure 6:**
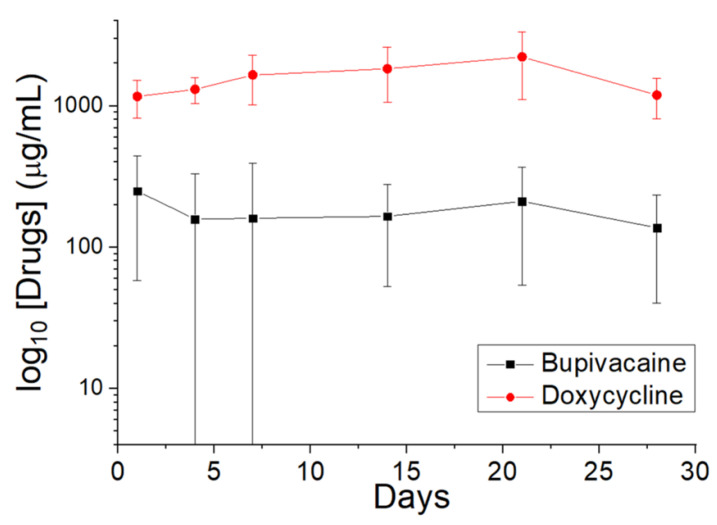
In vivo elution of doxycycline and bupivacaine from DCB–PLGA-composite nanofibers.

The in vivo discharge behavior (Figure 6) suggests that the DCB–PLGA-composite nanofibers can elute high levels of doxycycline and bupivacaine for 28 days in the tissues surrounding the implanted membranes.

### 3.4. Bioactivity and Water/Food Intake

**Figure 7:**
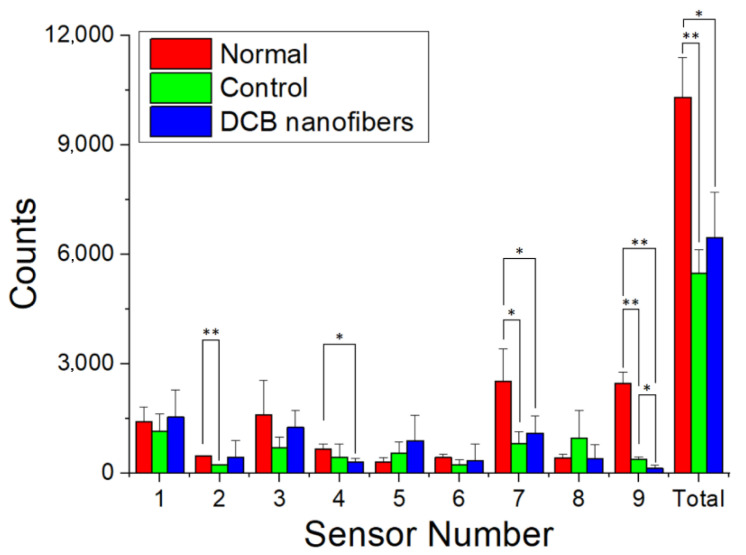
Bioactivity counts of the rats in various groups (* *p* < 0.05, ** *p* < 0.01).

The rats were housed within the ABC to examine their postoperative bioactivities. Figure 7 reveals that both the SD rats in the DCB-composite nanofibers and control groups (surgery only) exhibited inferior activity compared to the normal rats (no surgery). However, the rats implanted with the DCB nanofibers exhibited a greater activity count than the control rats, although the difference was insignificant (*p* > 0.05).

**Figure 8:**
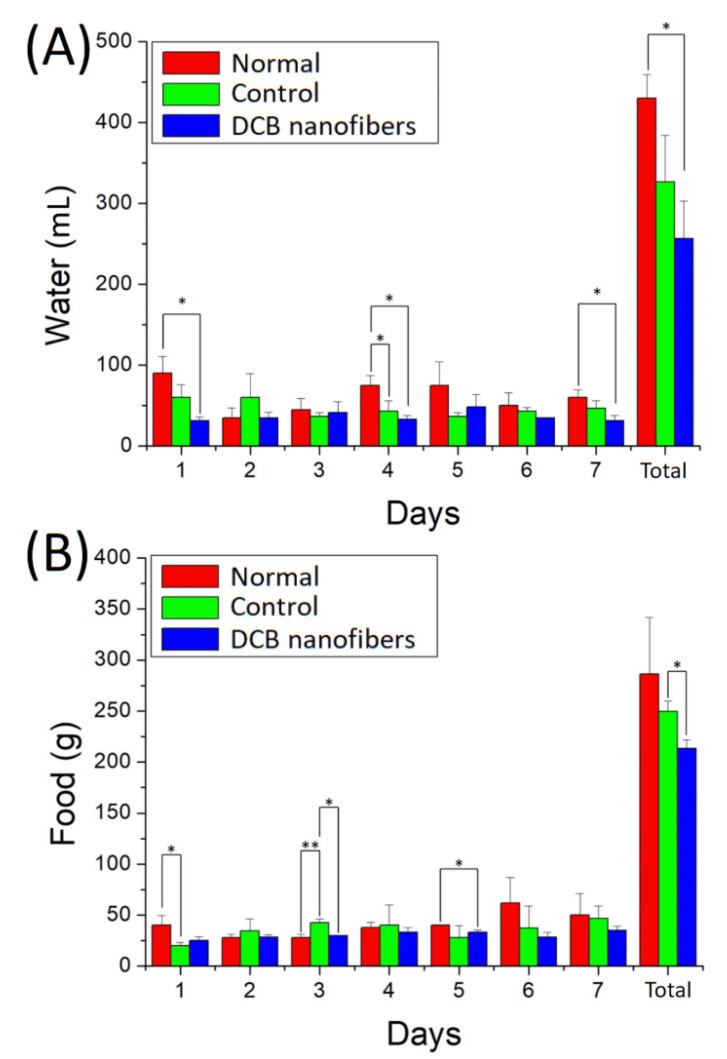
(**A**) Water, and (**B**) food intakes for the rats in various groups (* *p* < 0.05, ** *p* < 0.01).

Figure 8A,B show the water and food intake of the animals. The results suggest that the rats that received DCB nanofiber implantation consumed less water and food than those in the normal and control groups after the surgery.

### 3.5. Mechanical Property Assessment of Repaired Tendons

**Figure 9:**
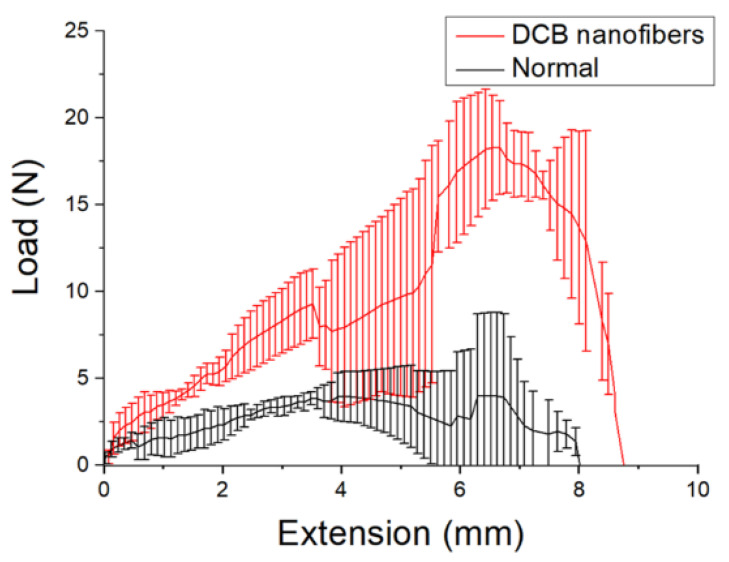
Tensile properties of normal tendons (maximum load-to-failure: 4.02 ± 4.74 N) and tendons repaired by the DCB–PLGA-composite nanofibers (maximum load-to-failure: 18.32 ± 2.69 N).

**Figure 10:**
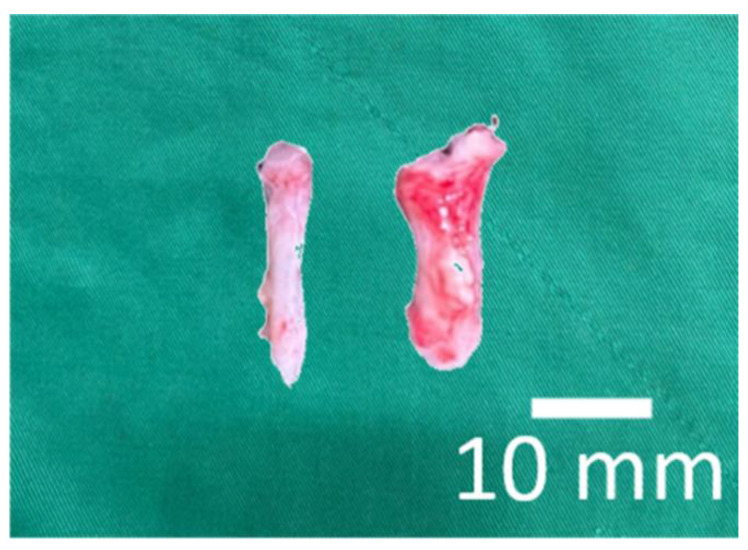
Photo of retrieved tendons (left: normal, right: repaired by the DCB–PLGA nanofibers).

Figure 9 shows the measured tensile strengths of the repaired tendons at eight weeks post-implantation. The DCB–PLGA-composite nanofibers repaired tendons exhibit significantly higher maximum load-to-failure (18.32 ± 2.69 N) than the normal tendons (4.02 ± 4.74 N) (*p* < 0.01). The experimental results demonstrate the nanofibers’ capability to repair ruptured tendons. This is mainly due to the larger diameter (5.2 ± 0.9 mm) of repaired tendons when compared to that of normal tendons (2.1 ± 0.4 mm), as shown in Figure 10.

### 3.6. Histological and Immunohistochemistry (IHC) Assessments

**Figure 11:**
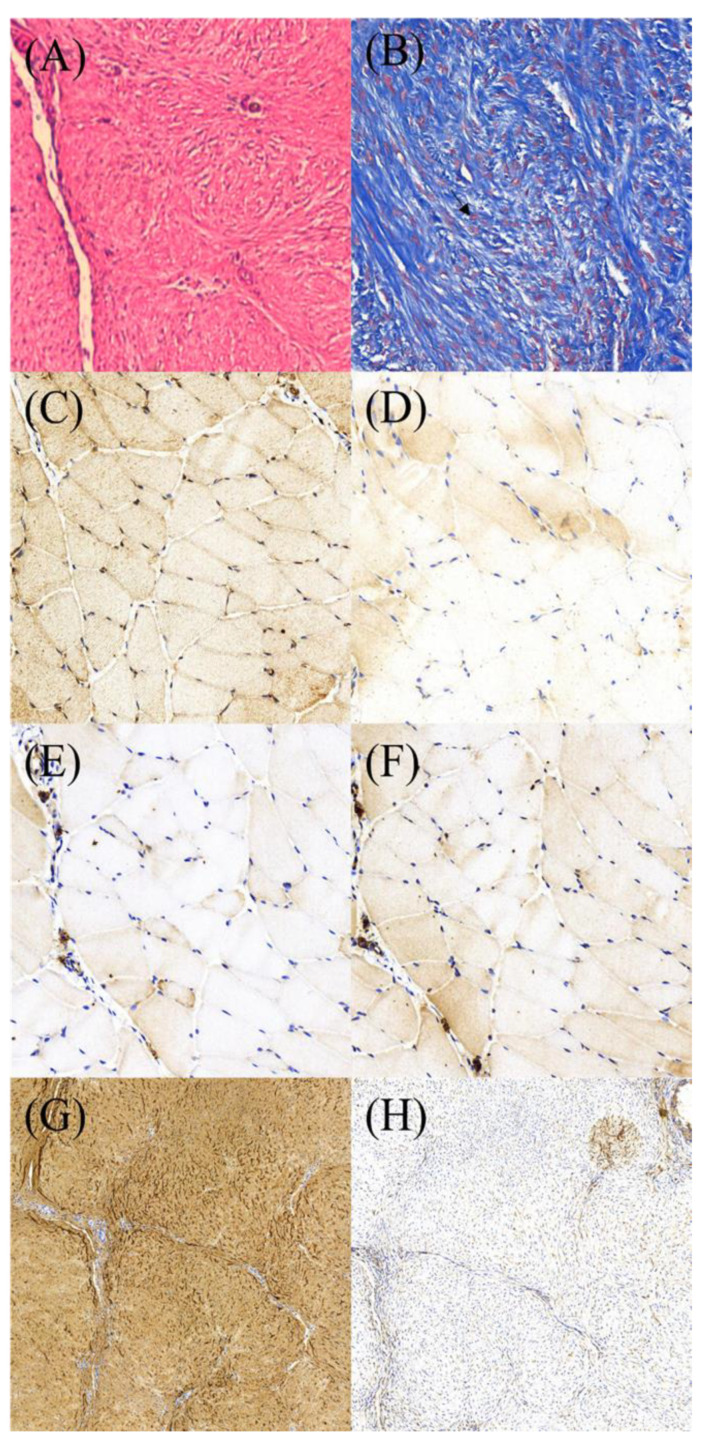
Photos of histology and IHC stain of the specimens from repaired tendons. (**A**) H&E stain (400×). (**B**) tenocytes (arrow) by Masson’s trichrome stain (400×). IHC stain for growth factors (800×): (**C**) VEGF, (**D**) BMP-2. (**E**) TGF-B, and (**F**) vWF. (**G**) and (**H**) showing collagen I and collagen III under IHC stain (400×).

HE staining revealed a regular organization of the collagens (Figure 11A). Abundant tenocytes in round or ovoid shapes (Masson’s trichrome stain) were scattered among the collages (Figure 11B). IHC staining demonstrated a strong cytoplasmic expression of VEGF and moderate expression of BMP-2, TGF-β, and VWF (Figure 11C–F). Figure 11G,H show that the average optical densities of collagen I and collagen III are 0.44 and 0.27, respectively, i.e., collagen I is significantly higher than collagen III (*p* < 0.05).

## 4. Discussion

Various treatments to repair Achilles tendon injuries beyond primary sutures have been reported [17,18,19]. A torn Achilles tendon can be nonsurgically or surgically treated. In general, an injured tendon undergoes a three-phase healing process: an early inflammatory phase, followed by proliferative and remodeling phases [31]. Although the injured tendon completes the remodeling, the healed tendon tends to have scar-like tissue that may not completely regain its original biomechanical properties [32,33]. The depletion of tenocytes and collagen I may be a significant reason for this property deterioration [34,35,36]. Therefore, in addition to surgical repair, researchers have advocated rebuilding the biological and physical properties of the tendon by upregulating the cellular and tissue responses during tendon repair, including the supplementation of bioactive growth factors, modulation of the inflammatory response, and adoption of tissue engineering [37,38,39,40,41].

Tissue engineering has been demonstrated as an effective approach for treating ruptured tendons. Liu et al. [42] developed collagen-incorporated PLGA nanofibrous scaffolds and showed that the scaffolds could accelerate healing at an early stage. Weng et al. [14] exploited resorbable doxycycline-loaded nanofibrous membranes and evaluated their effectiveness in treating ruptured Achilles tendons. Their results show that the doxycycline-treated rats exhibited comparable tendon strengths at 6 weeks post-operation to the rats in the control group (surgery only without drug treatment) and healthy rats (insignificant, *p* > 0.05).

In this study, DCB nanofibers were fabricated by integrating doxycycline, collage, and bupivacaine into PLGA nanofibrous membranes using an electrospinning technique. The experimental results illustrate that the strength of the repaired tendons is greater than that of normal tendons, demonstrating the superiority of DCB nanofibers over doxycycline-only nanofibers. Incorporating biomolecules into biodegradable nanofibers can stimulate various growth factors, and the injured Achilles tendon could possibly re-establish its biological composition and restore its mechanical strength.

Doxycycline falls into the category of the tetracycline antibiotic family and shows excellent activity against Gram-positive and Gram-negative microorganisms [43]. In addition to its well-proved antimicrobial capabilities, doxycycline inhibits phorbol-12-myristate-13-acetate-mediated matrix metalloproteinase 8 (MMP-8) and MMP-9 in human endothelial cells, reducing elastin degradation and MMP activity in a model of aneurysmal disease. The drug can also inhibit fibroblasts in epidermal scars and enhance collagen construction [44]. Conversely, bupivacaine acts as a local anesthetic in caudal, epidural, and spinal anesthesia by inhibiting NMDA receptor-mediated synaptic transmission in the dorsal horn of the spinal cord. This medicine has been clinically used for acute and chronic pain management.

PLGA nanofibers were acquired at a solution concentration corresponding to the creation of significant molecular chain entanglements in the polymers. The incorporation of collagen and pharmaceuticals reduces the content of PLGA in the solution and the solution viscosity. It becomes more difficult for the solution to resist the external electric force during electrospinning and the spun fiber size reduces accordingly.

In general, the release of biomolecules from the degradable nanofibrous mats comprises three stages: a primary peak release followed by diffusion- and degradation-governed release. Most of the loaded biomolecules were embedded in the bulk of the PLGA nanofibers during electrospinning. Nevertheless, the pharmaceuticals loaded on the surface of the nanofibers cause a primary peak. After an initial burst, the discharge behavior is mainly controlled by osmotic diffusion and polymeric degradation. In our study, a second discharge peak of doxycycline and bupivacaine was observed on day 7. Subsequently, a gradually diminishing profile was observed. In contrast, in the in vitro discharge profile, a sustained release of the loaded pharmaceuticals was observed without a primary release peak because in vivo metabolism is generally slower than in vitro metabolism, and the in vivo primary peak release was reduced.

Incorporating biomolecules into the PLGA nanofibers increased the wettability of the spun matrices, which in turn enhanced cell proliferation and tissue healing. Furthermore, the biomolecule-loaded PLGA nanofibrous mats had excellent extensibility and flexibility, demonstrating their feasibility as scaffolds to accommodate the extension/contraction of tendons in the therapeutic procedure.

Another advantage of DCB–PLGA nanofibrous scaffolds is the pain-control capability provided by eluted bupivacaine. Postoperative pain, especially in orthopedic surgeries, remains a major concern. Although systemic administration or local injection of analgesic agents is employed as a routine procedure after surgery, it has drawbacks, such as inadequate bioavailability or systemic adverse effects. [45,46] In this study, bupivacaine was incorporated into PLGA matrices as an analgesic and co-eluted with implanted PLGA nanofibrous scaffolds. The rats in the DCB nanofiber group showed an improved activity count compared to the rats in the control group, demonstrating that the analgesic function could effectively reduce postoperative pain and enable the rapid resumption of normal activities. Despite improved general activities, decreases in food and water consumption were also observed, and the loss of appetite was one of the side effects of high-concentration bupivacaine administration [47,48]. Further work is required to identify the optimum dose that provides appropriate pain relief with minimized side effects.

Despite the satisfactory outcomes of this study, there are some limitations. First, this animal study was conducted on normal Achilles tendons with sharp scalp-cut transections. However, a torn Achilles tendon in humans generally occurs in degenerated tendons after an unexpectedly distracted axial force. The tendon composition and healing process are slow or inadequate in degenerated torn tendons. Second, in vitro dose-dependent experiments on the loaded drugs were not performed. Further studies are recommended to elucidate the elution profiles of the drugs under different concentrations. Finally, the systemic concentrations of locally delivered drugs were not examined. Moreover, beyond the therapeutic effects of the drugs, side effects such as cardiac or renal involvement should also be assessed in the future.

## 5. Conclusions

We successfully established tri-layered multi-biomolecule-loaded composite biodegradable scaffolds for the repair of ruptured Achilles tendons. Doxycycline, collage, and bupivacaine were integrated into PLGA nanofibrous membranes as healing promoters using an electrospinning technique, 3D scaffold, and painkillers, respectively. The DCB nanofibrous scaffolds provided a sustainable release of loaded pharmaceuticals both in vitro and in vivo for clinical applications. Tendons treated with nanofibrous scaffolds exhibited a significantly greater maximum load-to-failure than normal tendons. Additionally, the composition of the healed Achilles tendon possessed abundant tenocytes and collagen I. The DCB–PLGA scaffolds show great potential for treating Achilles tendons in humans.

## Data Availability

All data generated and analyzed during this study are included in the published article.
